# Sea spray allows for the growth of subaerial microbialites at the driest desert on Earth

**DOI:** 10.1038/s41598-024-70447-x

**Published:** 2024-08-28

**Authors:** Armando Azua-Bustos, Carlos González-Silva, Kevin Freedman, Daniel Carrizo, Laura Sánchez-García, Miguel Ángel Fernández-Martínez, María Balsera-Manzanero, Victoria Muñoz-Iglesias, Maite Fernández-Sampedro, Thanh Quy Dang, Cristian Vargas-Carrera, Jacek Wierzchos

**Affiliations:** 1 Centro de Astrobiología (CAB), CSIC-INTA, 28850 Madrid, Spain; 2 Facultad de Ciencias, Universidad de Tarapacá, Arica, Chile; 3 University of California Riverside, Riverside, USA; 4 Departamento de Ecología, Facultad de Ciencias, Universidad Autónoma de Madrid y Centro de Investigación en Biodiversidad y Cambio Global (CIBC-UAM), Madrid, Spain; 5 Consultora ProBiota. E.I.R.L., Iquique, Chile; 6 Laboratoire de Planétologie et Géosciences, CNRS, LPG UMR 6112, Nantes Université, Univ Angers, Le Mans Université, 44000 Nantes, France; 7 Museo Nacional de Ciencias Naturales (CSIC), 28006 Madrid, Spain

**Keywords:** Microbiology, Environmental microbiology, Astrobiology

## Abstract

Due to its extreme conditions, microbial life in the Atacama Desert is known to survive in well-protected micro-habitats (hypolithic, endolithic, etc.), but rarely directly exposed to the environment, that is, epilithic habitats. Here we report a unique site, La Portada, a cliff confronting the Pacific Ocean in the Coastal Range of this desert, in which the constant input of water provided by the sea spray allows for the growth of a black-colored epilithic subaerial microbial ecosystem. Formed by a complex community of halophilic microorganisms belonging to the three domains of life, this ecosystem displays the typical three-dimensional structure of benthic microbialites, coherent with the presence of a diversity of cyanobacteria (including species from the genera that are known to form them), a constant high water activity and an ample availability of carbonate ions. From these microbialites we isolated *Hortae werneckii,* a fungal species which by producing melanin, not only explains the dark color of these microbialites, but may also play the role of protecting the whole community from extreme UV radiation. A number of biosignatures not only confirmed sea spray as the main source of water, but also suggests that one place to consider for the search of evidences of life on Mars would be on the paleo-coastlines that surrounded vanished oceans such as that on Aeolis Dorsa.

## Introduction

The Coastal Range is a mountain chain that runs along the coast of Chile at its western margin^1^. Although in the Atacama Desert the Coastal Range mostly falls precipitously into the Pacific Ocean, there are a few places containing narrow coastal plains such as the Mejillones Peninsula, where La Portada cliffs are located^2^. The Mejillones Peninsula is the most apparent irregularity along the otherwise near linear north Chilean coast, a large crustal block that consists of Paleozoic metamorphic rocks and Triassic–Jurassic granitoid rocks and basaltic dykes^3^. It is at the southern edge of this peninsula that we found a unique cliff containing a black-colored epilithic subaerial microbial ecosystem, with a three-dimensional structure characteristic of benthic microbialites (carbonate organo-sedimentary deposits that develop as a result of benthic microbial communities by trapping and binding detrital sediments)^4–6^, and composed by halotolerant/halophilic microorganisms. Culture dependent/independent analyses as well as biosignatures analyses unveiled the microbial members of these microbialites, but also showed that the point of origin of these species, as well as the main source of water, is the sea spray coming from the Pacific Ocean.

## Results and discussion

**Figure 1:**
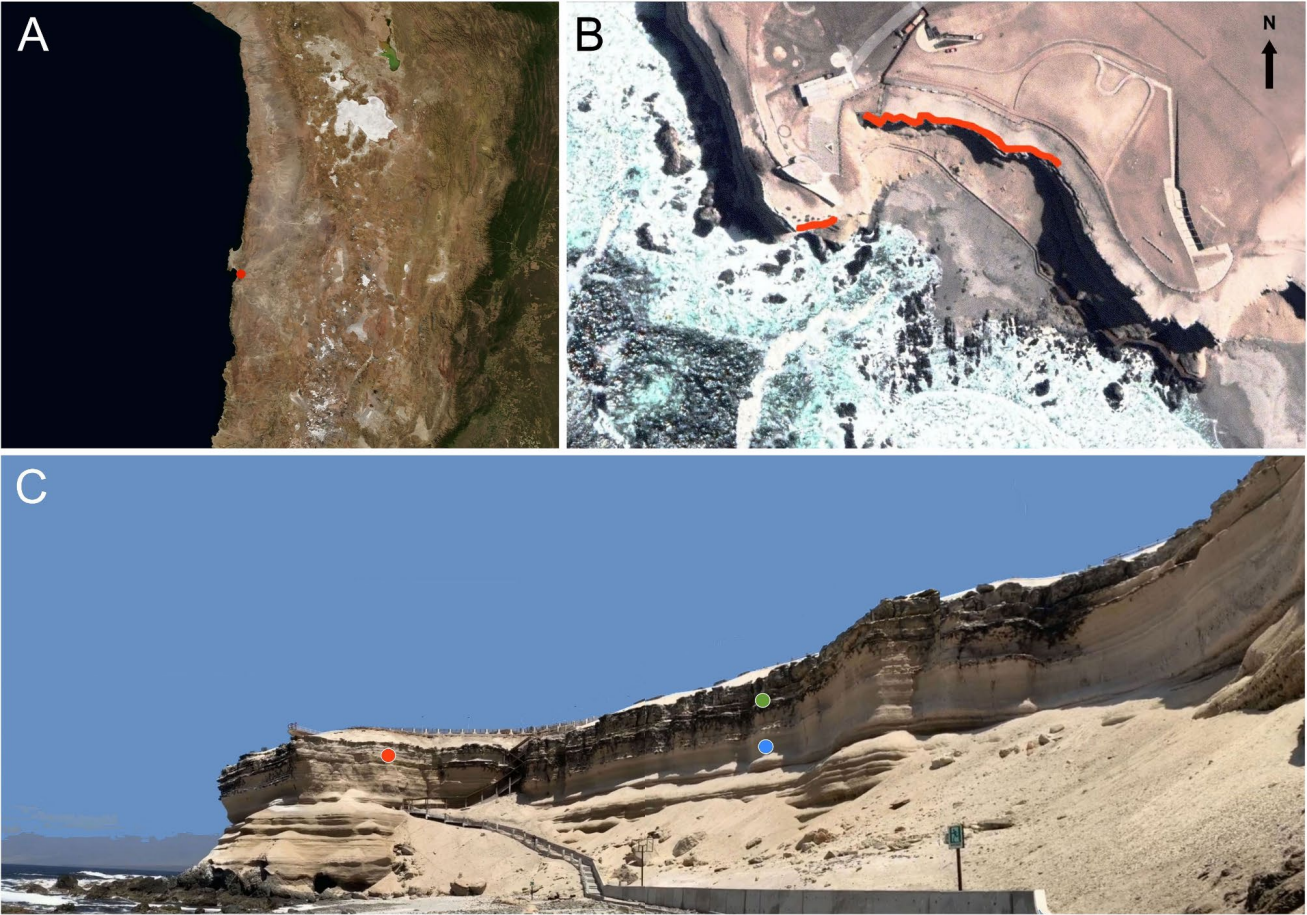
La Portada Cliffs location. (**A**) General location of the site (red spot) at the Coastal Range of the Atacama Desert. (**B**) A closer zenithal view of the site (Google Earth), in which the red lines show the south facing orientation where colonization takes place. (**C**) A view of the inspected site from the beach in front of it. The colored dots mark where temperature/RH sensors were embedded; red, East facing Uncolonized; green, South facing Colonized; light blue, South facing Uncolonized.

La Portada cliffs are located in northern Chile south of the coastal city of Antofagasta (Fig. 1A), directly confronting the Pacific Ocean (Fig. 1B). These cliffs, about 50 m high, are composed by a conglomerate bed of Miocene fossilized marine sandstone and fossil shells topping Jurassic breccias of basaltic andesite^7^ (Fig. 1C).

X-ray diffraction (Fig. S1, Table S1) and Raman spectroscopy (Fig. S2) showed the overlaying conglomerate to be composed of calcite (CaCO_3_), dolomite (CaMg(CO_3_)_2_) and albite (NaAlSi_3_O_8_), with minor amounts of halite, quartz, nimite, goethite and gypsum.

**Figure 2:**
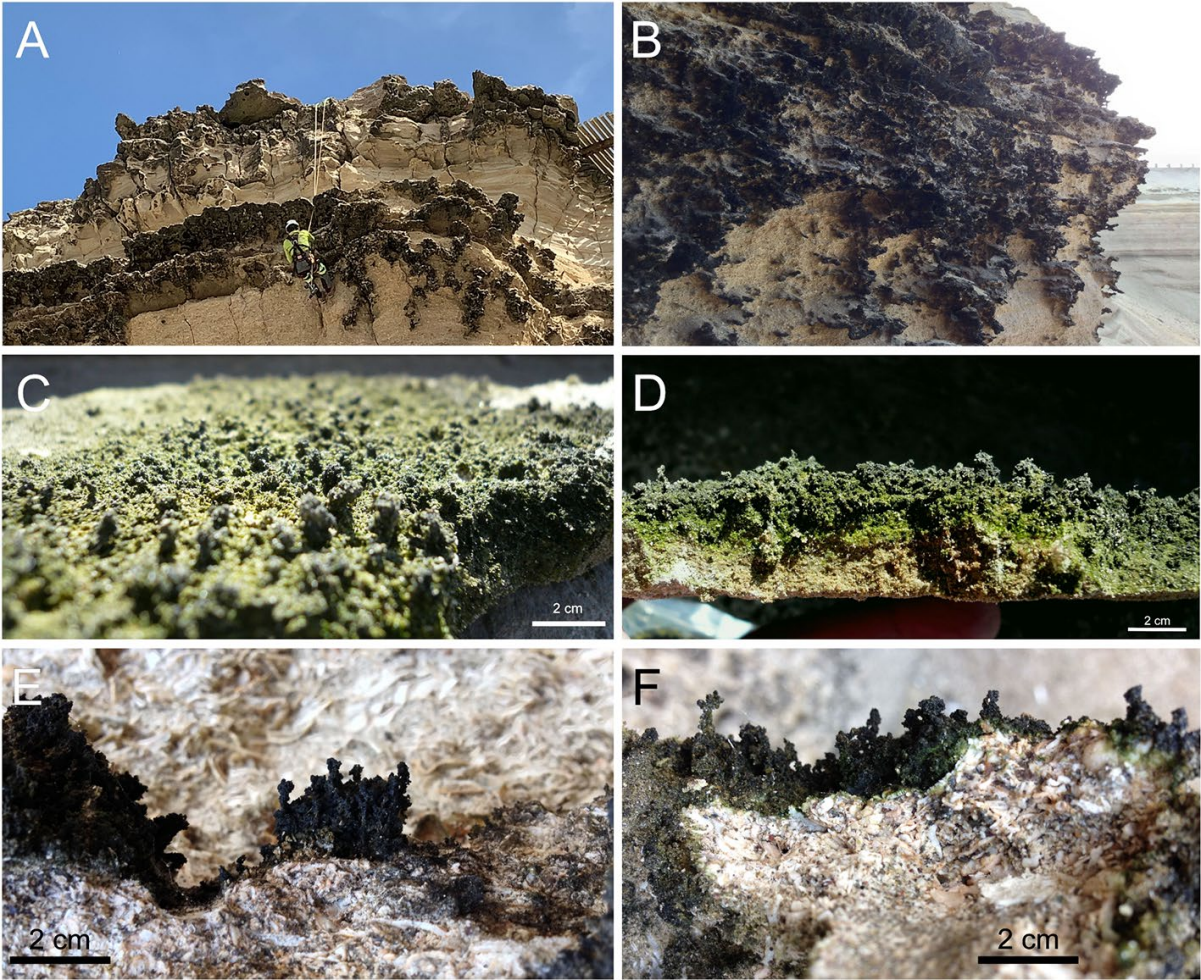
Close up of the colonized sections of La Portada cliffs. (**A**) A view of a portion of the colonized section while sensors were being embedded. (**B**) A closer view of the colonized section. (**C–F**) Show details of the finger-like structures common in the colonized sections of the cliffs.

Although in this area these cliffs run along for about 7 km, only the upper portions of a section of about 130 m of the overlaying conglomerate are colonized by a black-colored epilithic microbial layer (Fig. 2A,B), present only on the sections strictly facing south (Fig. 1B), which in some places reach up to four centimeters of thickness (Fig. 2C,D).

Relative humidity (RH) sensors embedded in these cliffs showed that RH reached up to 93.5% (equivalent to a water activity of 0.935) in the colonized zones (Figs. S3, S4A). Additional analyses showed that while in the uncolonized areas RH dropped down to 28.7% during the day (Fig. S4A), RH was consistently high in the south facing colonized cliffs, independent of the time of the day (Fig. S4B). Intriguingly, only the upper parts of the south facing cliffs are colonized (Fig. 1C). Thus, infrared thermography was used to unveil a difference of more than 20 °C between the colonized and uncolonized south-facing surfaces during daytime hours (Fig. S5), with temperatures reaching up to 44.8 °C in the uncolonized areas (Fig. S6). Considering that the highest UV radiation levels on Earth have been measured in the Atacama^8^, the absence of colonization despite the high RH present in the lower parts of the south facing cliffs may be explained as this section of the cliffs have a more favorable angle of exposure to the sun (Fig. 1C), as opposed to the inhabited sections of the cliffs that are vertical, thus not fully illuminated by the sun until late during the afternoon.

**Figure 3:**
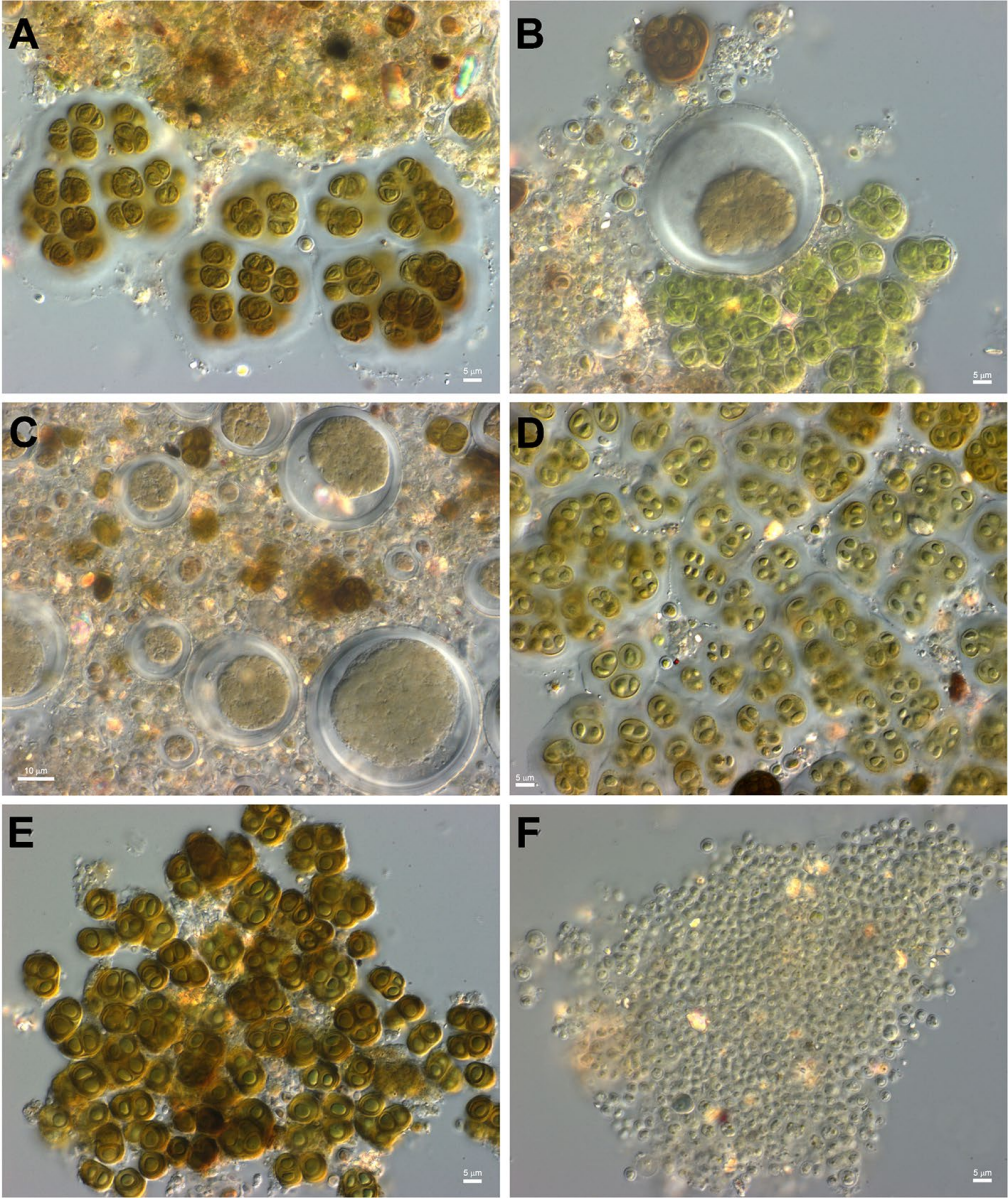
Bright field micrographs of La Portada microbialites. (**A–F**) Show the morphological diversity of cell types present, including different types of cyanobacteria, with most displaying thick exopolysaccharide envelopes.

A close inspection of the colonized areas showed small finger-like towers of cells (Fig. 2C–F) reaching a height up to two centimeters. Bright field microscopy showed a number of different microorganisms in these structures, including morphologically distinct cyanobacteria and bacteria (Fig. 3).

The use of culture dependent and independent methods allowed us to unveil the identity of the microorganisms present on the colonized areas. 16S/18S rRNA NGS metagenomic analyses showed a high diversity of microbial species representing the three domains of life (Fig. S7), with most OTUs (operational taxonomic units) phylogenetically close to bacterial, archaeal and eukaryotic species reported as halotolerant, (41.3%), i.e. *Halomonas*^9^, *Salinimicrobium*^10^, *Halomarina*^11^, *Saliniarchaeum*^12^, or halophilic (23.8%); *Alkaliphilus*^13^, *Halospina*^14^, *Haladaptatus*^15^, *Halococcus*^16^, *Hortaea*^17^, *Wallemia*^18^, including halotolerant (*Synechococcus*^19^) and halophilic (*Halothece*^20^) cyanobacteria.

The presence of OTUs belonging to ciliates (*Bistichella*), protists (*Gregarina*), amoeba (*Tulamoeba*), an even moss (*Syrrhopodon*), confirmed that water activity is high enough to even sustain the survival of such species under the harsh desiccating conditions of the driest desert on Earth.

NGS analyses also unveiled a number of the OTUs clearly belonging to marine species, such as mollusks (*Mytilisepta*), other marine invertebrates *(Pyura*), corals (*Zoanthus*), diatoms (*Synedra*), bacteria (*Jannaschia*) and marine macro algae (*Porphyra*) (Fig. S8). Considering that west to east winds coming from the Pacific Ocean are the principal mechanism of dispersal of microbial life into the Atacama^21^, along the high prevalence of halotolerant and halophilic microbial species in the colonized areas, NGS findings confirm that the main source of water for these microorganisms is the sea spray generated from the Pacific Ocean waves breaking in front of these cliffs (Fig. 1B,C).

The ample availability of carbonate ions in these cliffs (Figs. S1, S2), required for the nucleation of carbonate crystals on cyanobacterial exopolysaccharides^22^, the abundance of different species of cyanobacteria (Fig. 3, Fig. S7), including species such as *Halothece* and *Synechococcus* (known to be part of microbialites in saline benthic environments^23–26^) as well as the presence of gliding filamentous species such as *Limnofasciculus*^27^ (also known to be involved in the formation of modern coniform benthic microbialites^23^) (Fig. S7), suggest that the observed finger-like structures may be in fact fully subaerial microbialites (and in addition in the coast of the Atacama), in which these cyanobacteria mediate the in situ precipitation of carbonates, with its precipitation been similarly caused by cyanobacterial activity^28^ and/or evaporation^29^. If this is the case, this finding may be of importance for understanding the evolution of life on our planet, as all fossil microbialites are assumed to have been benthic^30^.

The use of a range of growth media allowed us to obtain a number of bacterial (*Oceanobacillus*, *Ornithinibacillus*, *Paenibacillus*, *Bacillus*) and fungal isolates (*Chaetomium*, *Sporormia*, *Aspergillus*)^31–35^, which coherent with the species found by NGS, are all halotolerant/halophilic^34,35–39^ (Fig. S9), and all reported in marine environments^32,37–40^ again coherent with NGS results.

**Figure 4:**
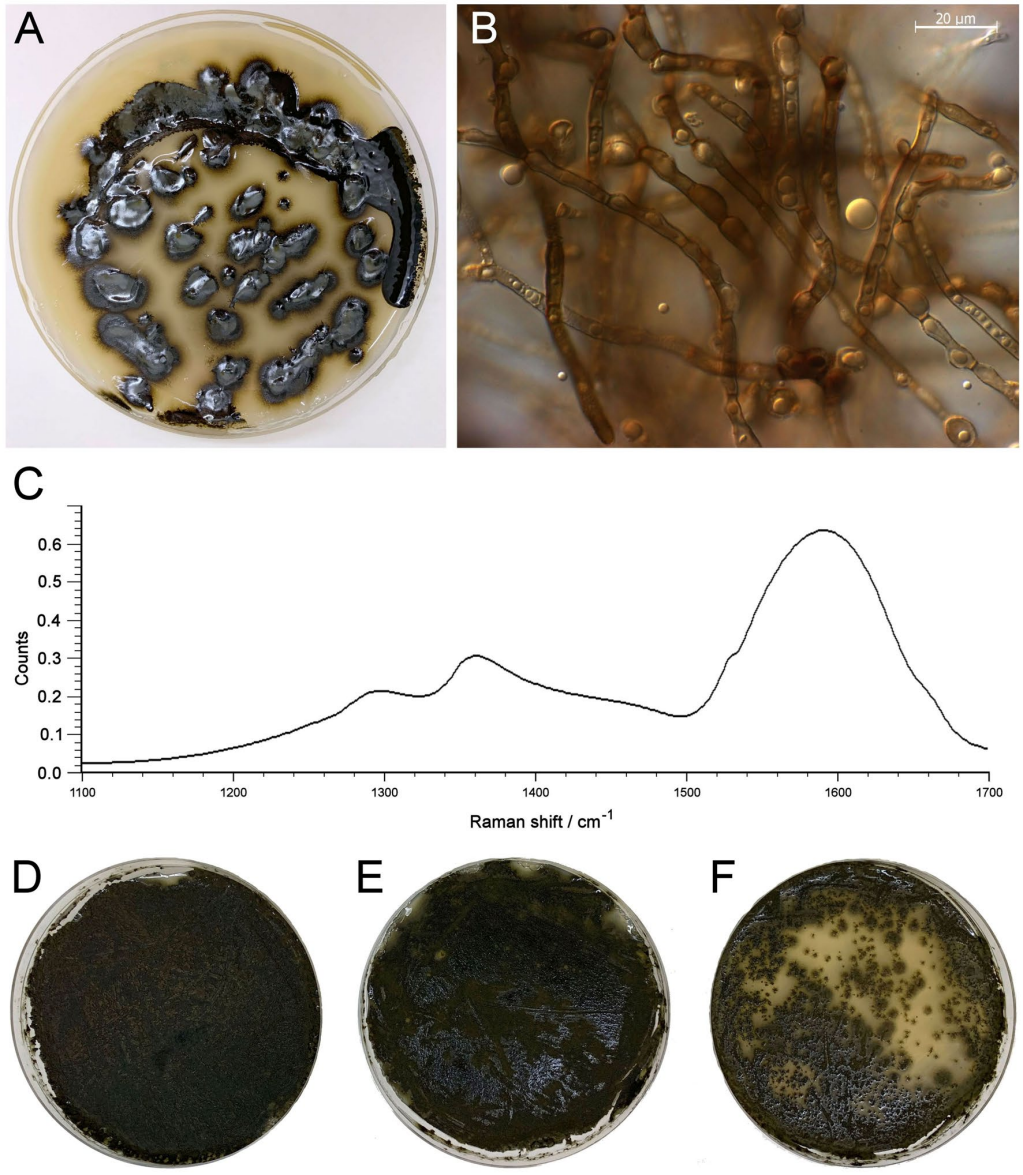
*Hortaea werneckii*, one of the fungal species isolated from the inspected microbialites. (**A**) Visual aspect of the isolate growing on a Petri dish. (**B**) Bright field micrograph of cells taken from the plate shown in (**A**). (**C**) Raman spectra of melanin isolated from the cells shown in (**A**). The typical Raman signal of fungal melanin consists of two main bands, with a higher intensity band located in the region 1590–1605 cm^−1^ and a second around 1350 cm^−1^ (Culka et al.^56^). (**D–F**) Aspect of the *H. werneckii* cells after irradiation for 10 min with UVA (**E**) and UVB (**F**). (**D**) Is the unexposed control. (**D–F**) show the plates 2 weeks after been irradiated.

Among the isolates found is *Hortaea werneckii* (Fig. 4A,B), (also detected by NGS, Fig. S7) a black-colored fungus previously reported inside a cave of the Coastal Range of the Atacama located 260 km further north^41^. *H. werneckii* is the most extremely halotolerant fungus known, and an important model organism for the study of halotolerance in Eukarya^17^. As the *H. werneckii* isolate found in La Portada synthetize important amounts of melanin^17^ (Fig. 4A,C), and for this reason extremely tolerant to UVA and UVB radiation (Fig. 4D–F), this fungus may play the role of protecting the subaerial microbialites from the extreme UV radiation typical of the Atacama^8^. Such role has been reported for other fungal species that are part of similar vertical finger-like structures in lichens under high sun irradiance in other desert environments^42^, in which protecting the photobionts allows for higher rates of CO_2_ assimilation.

Given that the Atacama Desert is a well-known analog model of Mars^43^, we also studied what type of biosignatures could be detected in the colonized areas. A variety of organics were detected (Tables S2, S3), with the most abundant (1.25 to 4.67 µg·g dw^−1^) being linear carboxylic acids (i.e. alkanoic acids) (Fig. S10), composed of chain lengths from C_14_ to C_22_, with C_16_ and C_18_ been the prevailing peaks. Other abundant compounds were mono-unsaturated alkanoic acids from C_16_ to C_20_ (0.01 to 0.21 µg·g dw^−1^) and terminally branched *iso*/*anteiso* alkanoic acids (i.e., linear carboxylic acids with a methyl group dangling from one of the last carbons of the chain) from C_14_ to C_18_ (0.001 to 0.52 µg·g dw^−1^). Alkanoic acids present in lower proportion were mid-chain branched carboxylic acids of 16 and 17 carbons with a methyl group in position C_10_. Linear alkanols of C_16_, C_17_ and C_18_ chain lengths were detected in the polar fraction, with concentrations ranging from 0.006 to 0.55 µg·g dw^−1^. In addition, unsaturated alkanols of C_16_ and C_18_ chain lengths, dihydrophytol and phytol (in concentrations from 0.005 to 0.287 µg·g dw^−1^) were also detected. The presence of acid lipids over the non-polar confirm the well-preserved biomass in the samples analyzed^44^, while dominant peaks at C_16_ and C_18_ of the linear acids, as well the abundances of unsaturated acids (C_16_, C_18_ and C_20_) and terminal branched fatty acids (iso/anteiso C_14_–C_18_) are consistent with a mayor contribution of bacteria^45^, coherent with culture dependent and independent biodiversity results.

In turn, the presence of dihydrophytol suggest again sea spray as the main source of water, as it is an intermediate in the biosynthesis of phytanic acid (synthesized from phytol^46,47^), the esterifying alcohol of chlorophyll C abundant in marine algae^48^.

Several of the aforementioned molecules (carboxylic acids^49^) have been studied as potential biosignatures to be searched for on Mars, with melanin being of particular interest, as it has been robustly detected by Raman spectroscopy and FTIR (Fourier Transform Infrared Spectroscopy) in samples exposed to Mars-like conditions outside the International Space Station^50^, thus detectable by the instruments that will be onboard the Rosalind Franklin rover of the future ESA’s ExoMars mission^51^.

Altogether, these findings show that a unique combination of environmental factors allow for the presence of subaerial epilithic microbialites containing halotolerant/halophilic members of the three domains of life in the coasts of the driest Desert on Earth, which main source of water, and origin, is the sea spray from the Pacific Ocean. In addition, as a number of biosignatures can be detected in these microbialites, the findings on this site suggest that an additional place to search for evidence of life on Mars would be on similar cliffs and surrounding landscapes of vanished oceans such as Aeolis Dorsa^52^.

## Methods

### Site sampling

La Portada site was first inspected in May of 2019, with microbialites and rock samples taken in the vertical profile of colonized and uncolonized sections of the cliffs. Further biological and rock samples were taken in October of 2019, December of 2019, May of 2022, July of 2022 and August of 2022. All samples were aseptically collected and stored in sterile falcon tubes, and stored at room temperature for further processing.

### X-ray diffraction

Powder X-ray diffraction of bulk rock samples was performed using a Bruker D8 Eco Advance with Cu Kα radiation and Lynxeye XE-T linear detector. Samples were scanned between 5° (2θ) and 60° (2θ) using a step size of 0.05° (2θ) and a count time of 1 s. The phase identification was performed by comparing the measured diffraction pattern with patterns of the PDF Database with the DIFFRAC.EVA software (Version 7, Bruker AXS, https://www.bruker.com/es/products-and-solutions/diffractometers-and-x-ray-microscopes/x-ray-diffractometers/diffrac-suite-software/diffrac-eva.html). Afterwards, the clay fraction was obtained by decantation according to Stokes’ law. The determination of clay involved treatment: air dried, solvation with ethylene glycol, and heating at 350 and 500 °C during two hours. Then Samples were scanned with a 0.02° (2θ) step size over the range 2°–30° (2θ) with a 1 s collection time at each step, all as previously performed by us^43^.

### Raman spectroscopy of mineral samples

Raman spectra were obtained exciting the sample with a non-polarized Nd: YAG solid state laser of 532 nm wavelength. After focusing onto a monochromator (Horiba Jobin Yvon HRi550), with a diffraction grating of 1200 grooves/mm, the scattered light was detected with a Charge Coupled Device (CCD), 1024 × 256 pixels, cooled to 203 K for thermal-noise reduction. The spectrometer, connected by fiber optics to a B&W Tek microscope with a 50× objective, allowed a spot size on the sample of 42 µm (Microbeam S. A., Spain). The spectral resolution, with slit width of 200 µm, results better than 5 cm^−1^. Raman spectra were taken at laser power of 50 mW, and 10–100 s of integration times and 1–3 accumulations, all as previously performed by us^43^.

### RH measurements

Temperature and relative humidity were measured by embedding dual iButton temperature/Humidity micro loggers (Maxim Integrated, San Jose, CA, USA) in the cliffs, as previously performed in other sites of the Atacama^43^, and set to take data every 30 min from May 2019 to July of 2022. Care was taken to leave the opening of the sensor parallel to the angle of the cliff, with no void space behind the sensor.

### Infrared thermography

Thermal images were captured using a camera in the afternoon of December of 2020 (summer in the southern hemisphere). Using the Fluke Connect software (Version 2.0.1.0, https://connect.fluke.com/en/stores/home), individual pixels (which store the temperature information of a picture taken with this camera) were then randomly selected from such images (three transects, with 10 pixels per transect) of colonized and uncolonized sections facing south and south-west.

### Bright field microscopy

Microbialites were scraped from its rocky substrate and suspended in milli-q water. Observations were made in differential interference contrast (DIC) using a Zeiss AXIO Imager M2 fluorescence microscope (Carl Zeiss, Jena, Germany) and an Apochrome × 60, n = 1.4 Zeiss oil-immersion objective. A CCD Axiocam HRc Rev 2 camera and AXIOVISION software (Version 4.7, Carl Zeiss, Oberkochen, Germany, https://www.micro-shop.zeiss.com/en/us/system/software-axiovision+software-products/1007/) were used to capture and record images.

### Microbialite DNA extraction

Microbialite samples were stored at room temperature and DNA extracted from them in the lab using the DNeasy PowerBiofilm Kit (Qiagen, Düsseldorf, Germany) according to the manufacturer instruction.

### Illumina NGS‑based 16S and 18S rRNA sequencing and analysis

DNA concentrations were quantified by Picogreen. Then, variable input of DNA and variable number of cycles were used in a first PCR with Q5® Hot Start High-Fidelity DNA Polymerase (New England Biolabs) in the presence of: 100 nM primers for 16S amplification (5ʹ-ACACTGACGACATGGTTCTACACCTACGGGNGGCWGCAG-3ʹ and 5ʹ-TACGGTAGCAGAGACTTGGTCTGACTACHVGGGTATCTAATCC-3ʹ, these primers amplify the V3–V4 region of 16S), 200 nM primers for 18S amplification (5ʹ-ACACTGACGACATGGTTCTACAGCCAGCAVCYGCGGTAAY-3ʹ and 5ʹ-TACGGTAGCAGAGACTTGGTCTCCGTCAATTHCTTYAART-3ʹ), 200 nM primers for Archaea amplification (5ʹ-ACACTGACGACATGGTTCTACACGGRAAACTGGGGATAAT-3ʹ and 5ʹ-TACGGTAGCAGAGACTTGGTCTTRTTACCGCGGCGGCTGBCA-3ʹ), 200 nM primers in the case of ITS (5ʹ-ACACTGACGACATGGTTCTACATCCTCCGCTTATTGATATGC-3ʹ and 5ʹ-TACGGTAGCAGAGACTTGGTCTGTGAATCATCGAATCTTTGAA-3ʹ) and 200 nM primers for Cyanobacteria (5ʹ-ACACTGACGACATGGTTCTACAGGGGAATYTTCCGCAATGGG-3ʹ as Forward, and 5ʹ-TACGGTAGCAGAGACTTGGTCTGACTACTGGGGTATCTAATCCCATT-3ʹ or 5ʹ-TACGGTAGCAGAGACTTGGTCTGACTACAGGGGTATCTAATCCCTTT-3ʹ as Reverse). After the first PCR, a second PCR of 12 or 14 cycles was performed with Q5® Hot Start High-Fidelity DNA Polymerase (New England Biolabs) in the presence of 400 nM of primers (5ʹ-AATGATACGGCGACCACCGAGATCTACACTGACGACATGGTTCTACA-3ʹ and 5ʹ-CAAGCAGAAGACGGCATACGAGAT-[10 nucleotides barcode]-TACGGTAGCAGAGACTTGGTCT-3ʹ) of the Access Array Barcode Library for Illumina Sequencers (Fluidigm). The obtained amplicons were validated and quantified by Bioanalyzer and an equimolecular pool was purified by agarosa gel electrophoresis and titrated by quantitative PCR using the “Kapa-SYBR FAST qPCR kit forLightCycler480” and a reference standard for quantification. The pool of amplicons were denatured prior to be seeded on a flowcell at a density of 10 pM, where clusters were formed and sequenced using a “MiSeq Reagent Kit v3”, in a 2 × 300 pair-end sequencing run on a MiSeq sequencer.

Raw sequences were processed in MOTHUR software (v.1.43.0, https://mothur.org/) using a custom script based upon MiSeq SOP that maximizes sequence accuracy by restrictive quality thresholds at several steps, as previously and broadly employed before^43^. The resulting identity sequences were then manually checked using the Megablast option for highly similar sequences (only > 95% sequence similarity was accepted) of the BLASTN algorithm against the National Centre for Biotechnology Information nonredundant database (www.ncbi.nlm.nih.gov), all as previously performed by us^43^.

### Isolation of microbialite microorganisms

In the lab, samples were stored at room temperature and then inoculated in Petri dishes containing agar and either Luria–Bertani Broth (Sigma-Aldrich, Missouri, USA), Marine Media or modified Czapek Dox growth media (CondaLab, Torrejón de Ardoz, Spain). This was done by directly sprinkling 100 mg per sample into the Petri dishes containing the aforementioned media, and incubating these plates at 25 °C. In most cases colonies arising from the samples were evident 2 weeks after of inoculation. Colonies were separated and then re-cultivated in the media from they were first isolated, in order to obtain enough biomass for subsequent DNA extraction and storage, all as previously performed by us^43^.

### DNA extraction from isolates

DNA from the isolates was obtained from them in the lab using the DNeasy UltraClean Microbial Kit (Qiagen, Düsseldorf, Germany) according to the manufacturer instructions.

### Isolate identification

As previously performed^43^, 16S rRNA genes of bacterial isolates were amplified in the lab using the GoTaq Green Master Mix (Promega, Wisconsin, USA) and the primers 341f (5′CCT ACG GGNGGC WGC AG3′) and 785r (5′GAC TAC HVGGG TAT CTA ATC C′). PCR conditions used were: 95 °C for 5 min, and 25 cycles of (95 °C for 40 s, 55 °C for 2 min, 72 °C for 1 min) followed by 72 °C for 7 min. Similarly, 18S rRNA of eukaryotic isolates were amplified in the lab using the GoTaq Green Master Mix (Promega, Wisconsin, USA) and the primers F566 (5ʹCAG CAG CCG CGG TAA TTCC3′) and R1200 (5ʹCCC GTG TTG AGT CAA ATT AAG C3′). PCR conditions used were: 95 °C for 15 min, and 35 cycles of (95 °C for 45 s, 60 °C for 45 s, 72 °C for 1 min) followed by 72 °C for 10 min. The resultant reactions were visualized in a 2% agarose TAE gel at 50 V. The automated sequencing of the resulting PCR products was conducted by Macrogen DNA Sequencing Inc. (Seoul, Korea). Sequences were checked for quality using the BioEdit software (Version 5.0.9, https://thalljiscience.github.io/) and end-trimmed before using the Megablast option for highly similar sequences of the BLASTN algorithm against the National Centre for Biotechnology Information nonredundant database (www.ncbi.nlm.nih.gov) to search for the closest species of each of the isolates obtained. Phylogenetic analysis of 16S rRNA and 18S rRNA isolate gene sequences was performed by aligning sequences by multiple sequence comparison by log-expectation, analyzed with jModelTest and then by Phylip NJ (bootstrap 10,000), all tools of the freely available Bosque phylogenetic analysis software (version1.7.152, http://bosque.udec.cl).

### *Hortaea werneckii* growth conditions

*H. werneckii* was isolated as detailed in Zalar et al.^17^, and grown in Malt Extract Agar media (MEA; 20 g Malt Extract, 1 g Peptone, 20 g Glucose per liter), containing 5% of NaCl, with a pH adjusted at 5.5. Plates were incubated at 25 °C.

### *Hortaea werneckii* melanin extraction

Melanin from the *H. werneckii* was extracted by mixing 500 mg of cells with 5 ml 1 N NaOH and autoclaving at 121 °C for 30 min. The resulting solution was centrifuged at 5000 rcf for 5 min to remove cell debris. The supernatant was collected and acidified using 1N HCL until its pH reached 1.8–2. The acidified mixture was incubated at RT overnight to allow for melanin precipitation. The precipitate was then collected by centrifugation at 12,000 rcf for 20 min, and washed (resuspended and centrifuged for 20 min) thrice with distilled water. The resulting pellet was air dried overnight and stored at room temperature.

### *Hortaea werneckii* UV exposure

50 mg of *H. werneckii* cells were spread until they were not visible into triplicate Petri dishes containing MEA media, and separately exposed to UVA and UVB for 10 min, for a total of 2.42 J/cm^2^ (4 mW/cm^2^) and 5.6 J/cm^2^ (9.2 mW/cm^2^) respectively. After exposure, plates were incubated at 25 °C for 2 weeks.

### Raman characterization of melanin samples

Raman spectra of the extracted melanin was obtained using an inVia Qontor raman microscope with a 532 nm laser at 5 mW output power. The shown data was obtained by compiling 10 acquisitions, each one comprised of 10 accumulations, 10 s long. Data was passed through a noise filter, smoothed and baseline corrected using the WiRE™ software (version 5.6., https://www.renishaw.com/en/raman-software--9450).

### Lipid extraction, fractionation and analysis

Lipids were extracted according to the combination of methods as previously described^53,54^, modified to use small solvent volumes^55^. Lyophilized subsamples (~ 6 g), were spiked with internal standards (tetracosane-D_50_, 2-hexadecanol and myristic acid-D_27_) and then extracted with a mixture of dichloromethane and methanol (DCM:/MeOH, 3:1, v/v) by ultrasound sonication (3 × 10 min cycles). The total lipid extract (TLE) was concentrated to ~ 0.5 ml using rotary evaporation and then digested in a mixture (2 ml) of methanolic potassium (6% w/w) one hour at 100 °C, further separated into neutral and acidic fractions. The neutral lipid fraction was obtained by extracting the methanolic potassium mixture with 2 ml of *n*-hexane (Hx) three times, evaporating and recovering it with ~ 0.5 ml of Hx:DCM (9:1, v/v). The acidic lipid fraction was obtained by adding HCl to the remaining methanolic potassium mixture and extracting it with 2 ml of Hx (three times), then it was concentrated until 1 ml. Further separation of the neutral fraction into non-polar (hydrocarbons) and polar sub-fractions was done by eluting the concentrated neutral fraction (~ 0.5 ml of Hx:DCM) on an alumina column using ~ 0.5 g of Al_2_O_3_ powder in a precombusted Pasteur pipet with 4 ml of Hx:DCM (9:1, v/v) and 3 ml of DCM:methanol (1:1, v/v), respectively. All fractions (non-polar, polar and acid) were analyzed by gas chromatography mass spectrometry (GC–MS), by direct injection on Hx (apolar), meanwhile both the acidic and polar fractions needed to be derivatized with methanolic BF_3_ and BSTFA, respectively, as detailed elsewhere^54^, to transform the fatty acids into methyl esters (FAME) and the alcohols into trimethyl silyl derivates before analysis. No derivatization step was needed for the non-polar fraction. GC–MS analysis was made using an 8860 GC system coupled to a 5977B MSD (Agilent Technologies) operating with electron ionization at 70 eV and scanning from *m/z* 50 to 650. 1 µl of analytes were injected and separated on a HP-5MS column (30 m × 0.25 mm i.d. × 0.25 µm film thickness) with He as a carrier gas at a constant flow of 1.1 ml/m. All fraction were analyzed using the same oven temperature program, starting at 50 °C (held 1.5 min), then gradually increase to 130 °C at 20 °C/min and then to 310 °C at 6 °C/min (held 20 min). The injector temperature was set at 290 °C, the transfer line was at 300 °C and the MS source at 240 °C. Compounds identification was based on the comparison of mass spectra with reference materials, and their quantification on the use of external calibration curves of *n*-alkanes (C_10_ to C_40_), alkanols (C_14_ to C_24_) and fatty acid methyl esters (FAME; C_8_ to C_24_). All chemicals and standards were supplied by Sigma Aldrich (San Luis, Missouri, USA). The recovery of the internal standards averaged 77 ± 11%.

### Supplementary Information

Supplementary Information.

## Data Availability

All data and samples mentioned in this report are freely available upon request to AAB.
