# Supplementary material for: Sea spray allows for the growth of subaerial microbialites at the driest desert on Earth
Source: Sci Rep. 2024 Aug 28;14:19915. doi: 10.1038/s41598-024-70447-x (PMC11358262; doi:10.1038/s41598-024-70447-x)
Supplement: Supplementary file 1 — Supplementary Information. [file 41598_2024_70447_MOESM1_ESM.docx]

**Sea spray allows for the growth of subaerial microbialites at the driest desert on Earth**

**Authors**

Armando Azua-Bustos^1*^, Carlos González-Silva^2^, Kevin Freedman^3^, Daniel Carrizo^1^, Laura Sánchez-García^1^, Miguel Ángel Fernández-Martínez^4^, María Balsera^5^, Victoria Muñoz-Iglesias^1,6^, Maite Fernández-Sampedro^1^, Thanh Quy Dang^5^, Cristian Vargas-Carrera^5^, Jacek Wierzchos^7^.

**Affiliations**

^1^Centro de Astrobiología (CSIC-INTA), 28850 Madrid, Spain.

^2^Facultad de Ciencias, Universidad de Tarapacá, Chile

^3^University of California Riverside

^4^Departamento de Ecología, Facultad de Ciencias, Universidad Autónoma de Madrid y Centro de Investigación en Biodiversidad y Cambio Global (CIBC-UAM)

^5^Consultora probiota. E.I.R.L., Iquique, Chile

^6^Nantes Université, Univ Angers, Le Mans Université, CNRS, Laboratoire de Planétologie et Géosciences, LPG UMR 6112, 44000 Nantes, France

^7^Museo Nacional de Ciencias Naturales (CSIC), 28006 Madrid, Spain.

**Supplementary Files**


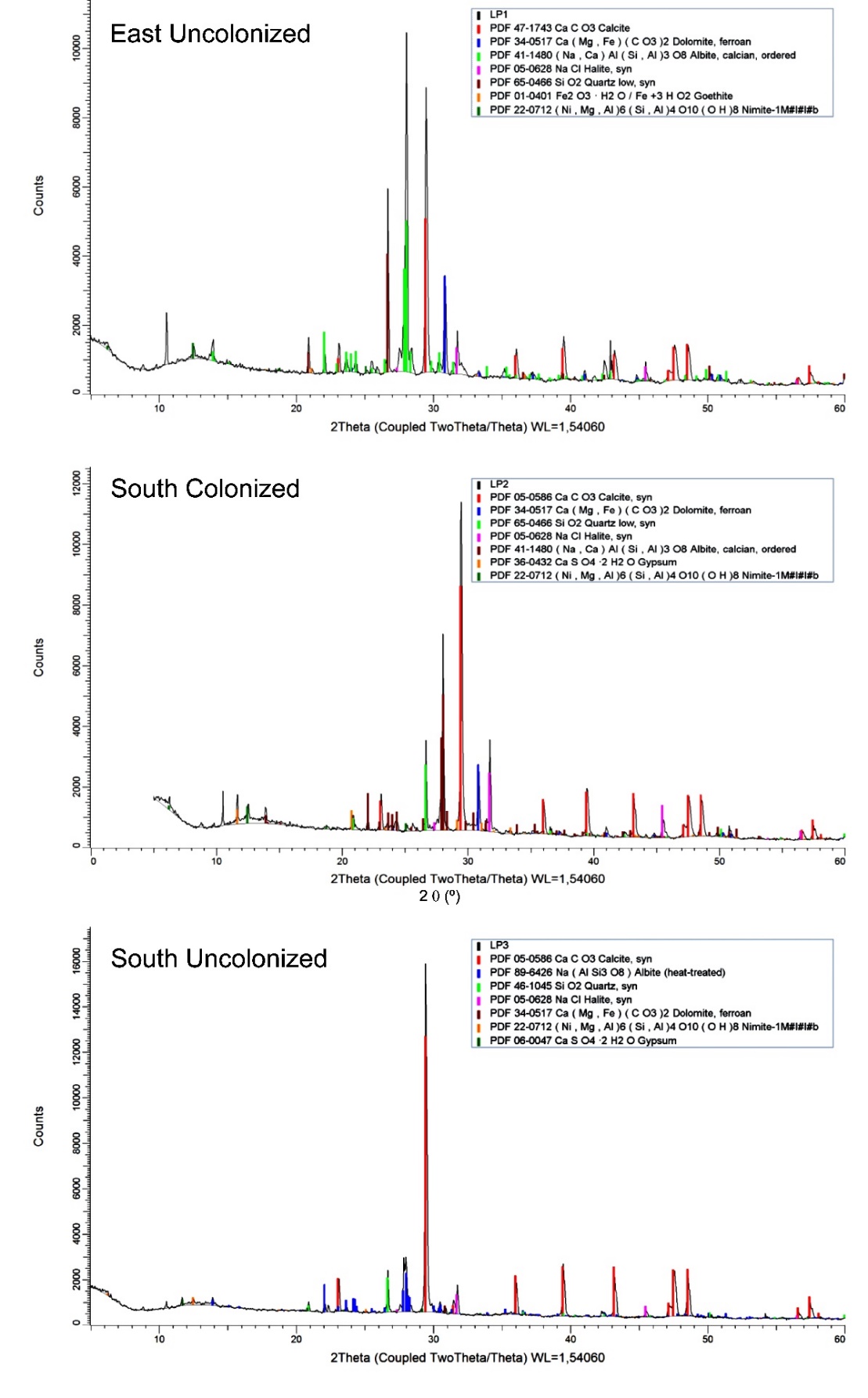


**Figure S1**. X-Ray diffraction (XRD) patterns of La Portada cliff rock samples.


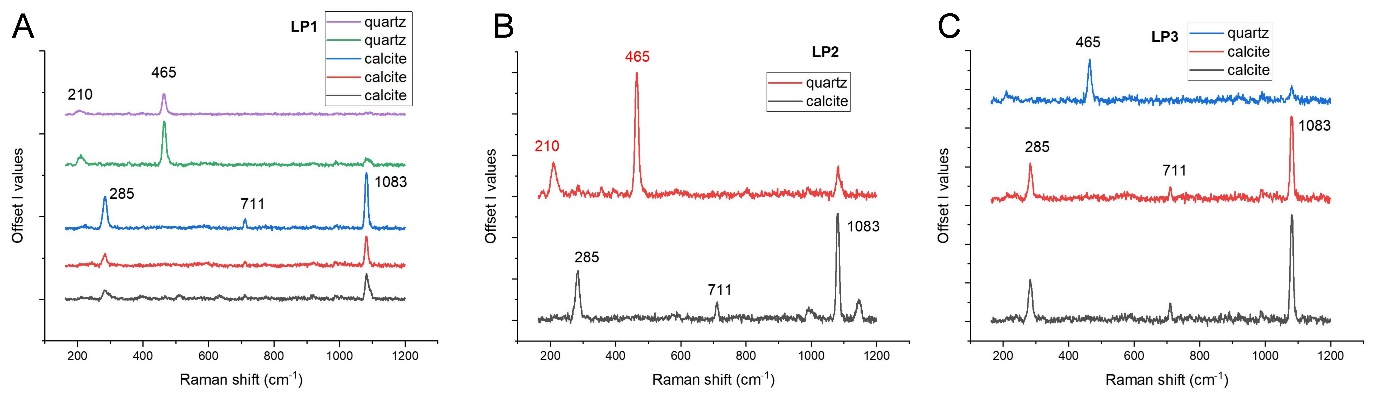


**Figure S2.** Raman spectra of La Portada cliff rock samples. A) Rocks of the uncolonized section of the cliffs (red dot in Figure 1C). B) Rocks samples taken 10 cm underneath the south facing colonized section of the cliffs (green dot in Figure 1C). C) Rocks of the south facing uncolonized section of the cliffs (light blue dot in Figure 1C).


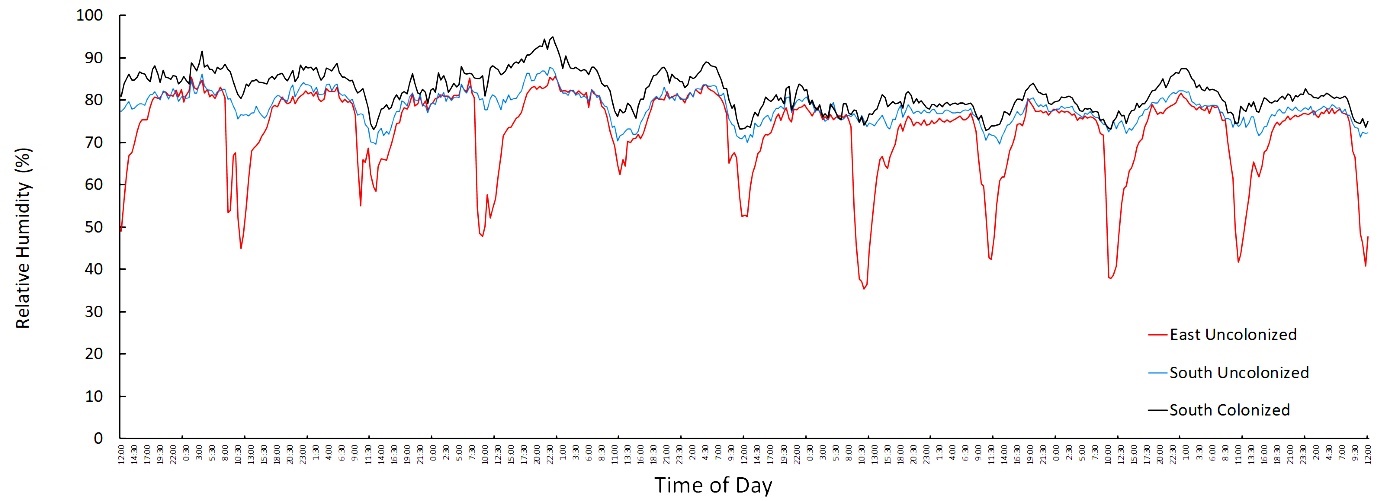


**Figure S3**.- Representative (10 days) relative humidity conditions at La Portada cliffs. Ticks in the x axis mark 12-h periods.


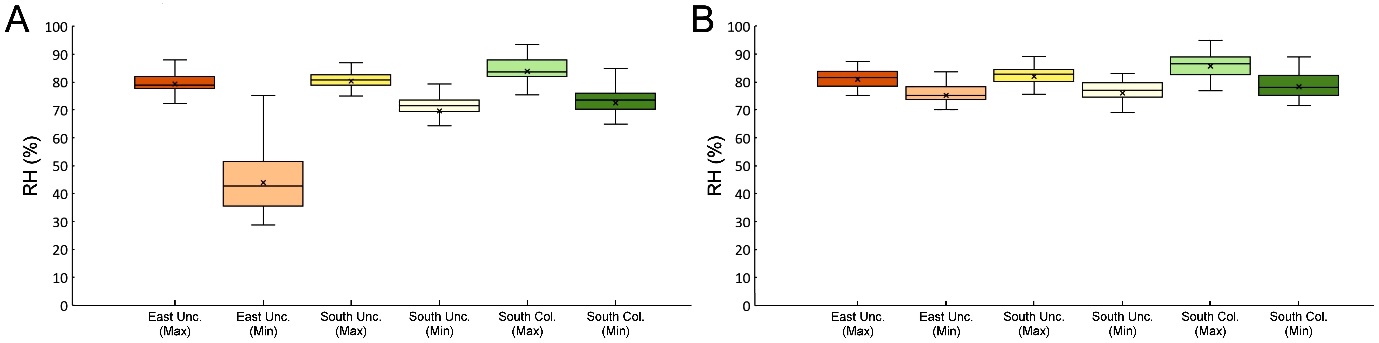


**Figure S4**. Box plot analyses of day and night time maximum and minimum RH at La Portada cliffs. A, day time hours. B, night time hours.


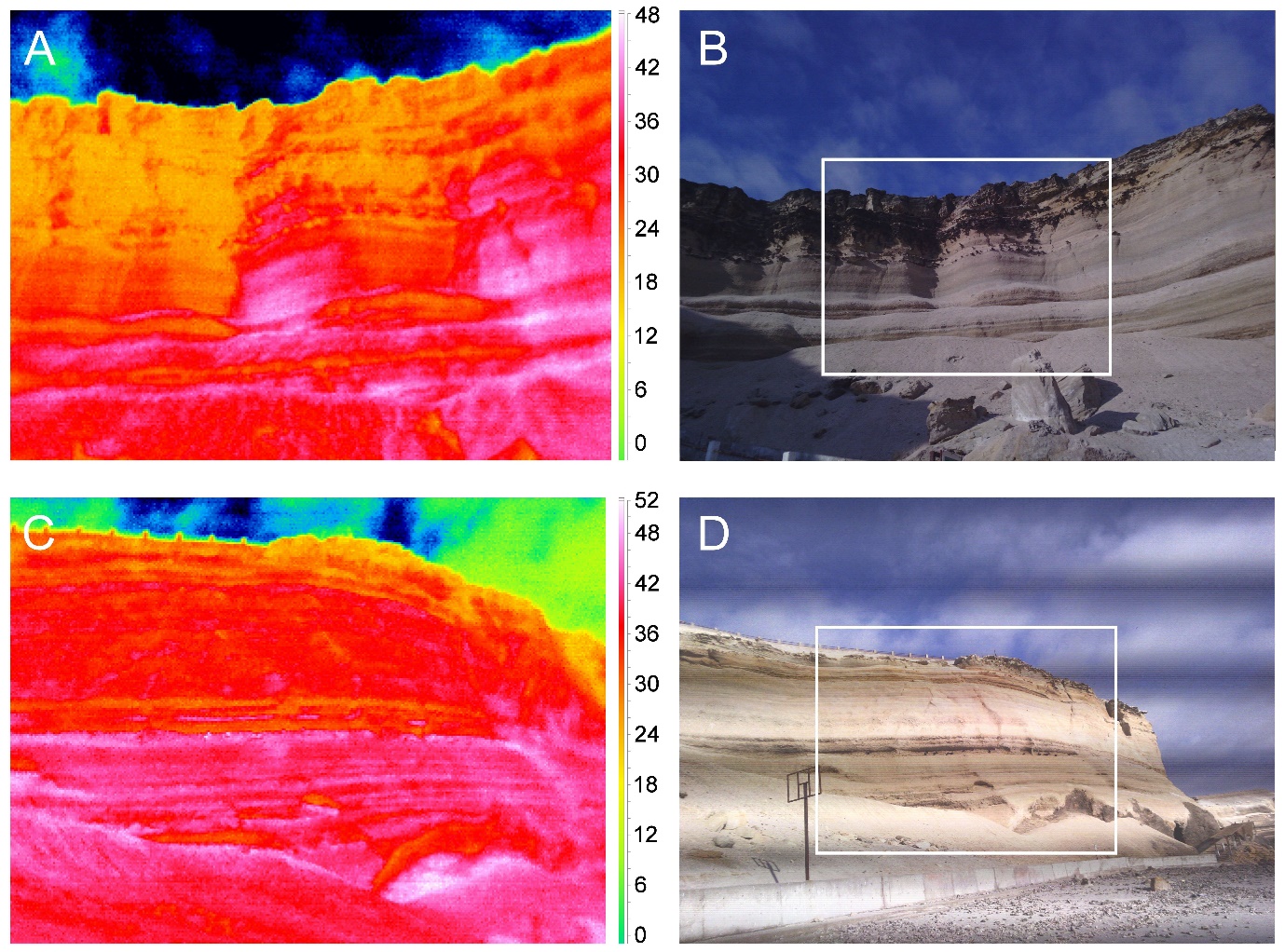


**Figure S5**. Infrared thermal images of La Portada cliffs. A, infrared image of inset shown in B, focused in the south facing colonized areas of the cliffs. C, infrared image of inset shown in D, focused in the south-west uncolonized areas of the cliffs. Temperature scales are in ºC.


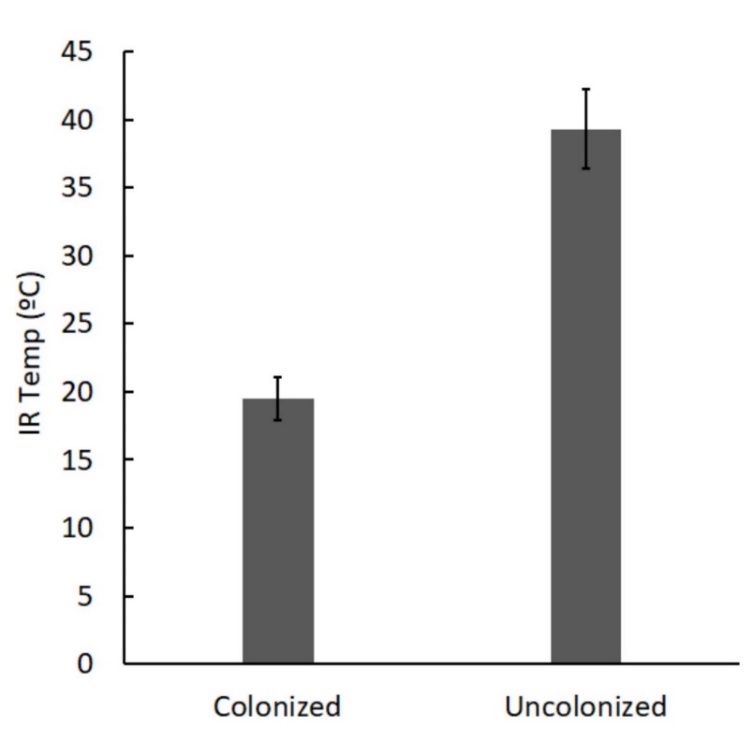


**Figure S6**. Infrared thermal differences between colonized (panel B of figure S5) and uncolonized areas (panel D of figure S5) of La Portada cliffs. Temperatures were measured and analyzed as detailed in methods.


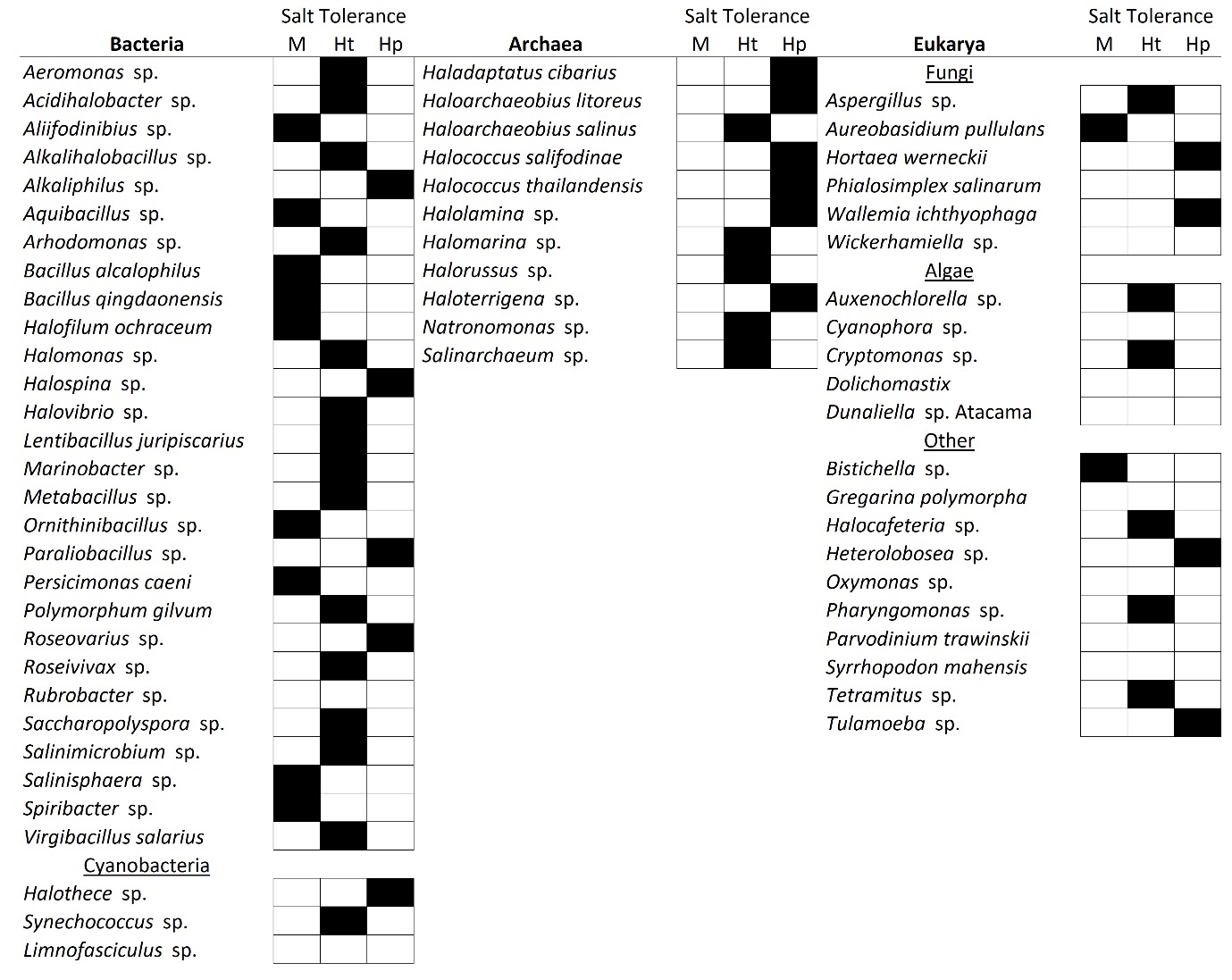


**Figure S7**. OTUs of microbial species detected in La Portada cliffs microbialites by Illumina NGS‑based 16S and 18S rRNA Sequencing. M: minor Halotolerance (17,5%). Ht: halotolerant (41,3%). Hp: Halophile (23,8%). White cells show species which haloterance has not been reported (17,5%).


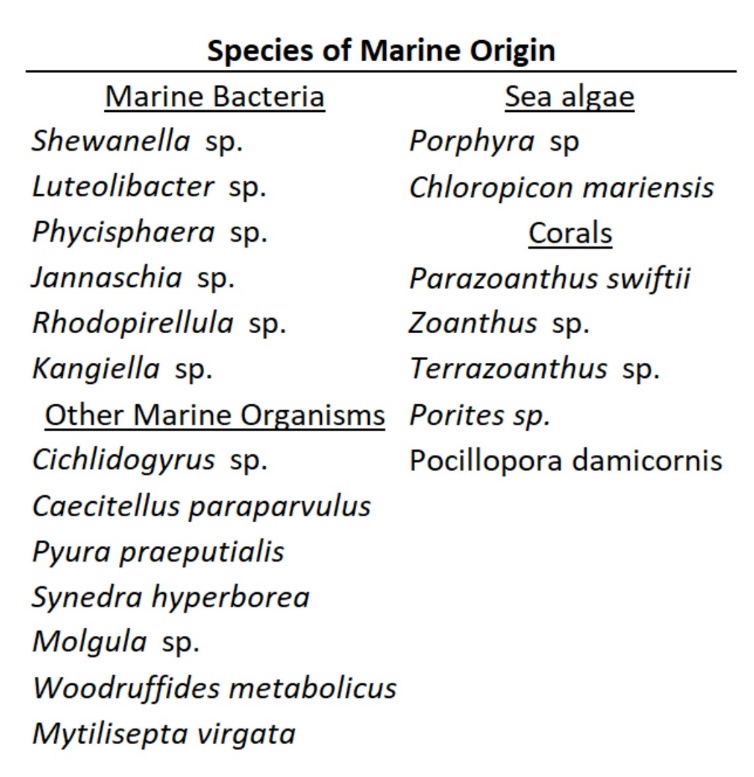


**Figure S8**. OTUs of marine species detected by Illumina NGS‑based 16S and 18S rRNA Sequencing in La Portada cliffs microbialites.


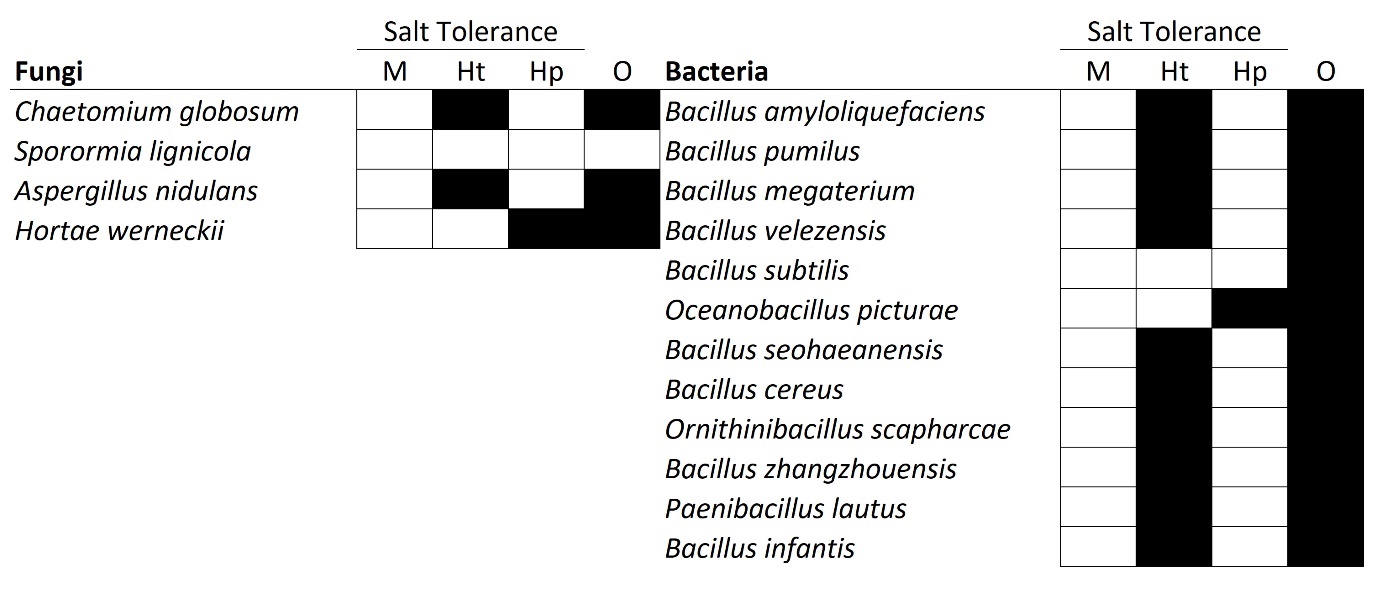


**Figure S9**. Microbial isolates obtained from La Portada cliffs microbialites. M: minor Halotolerance (0%). Ht: halotolerant (75%). Hp: Halophile (12,5%), not reported (white cells, 12,5%). O: species reported in oceans (93,75%).


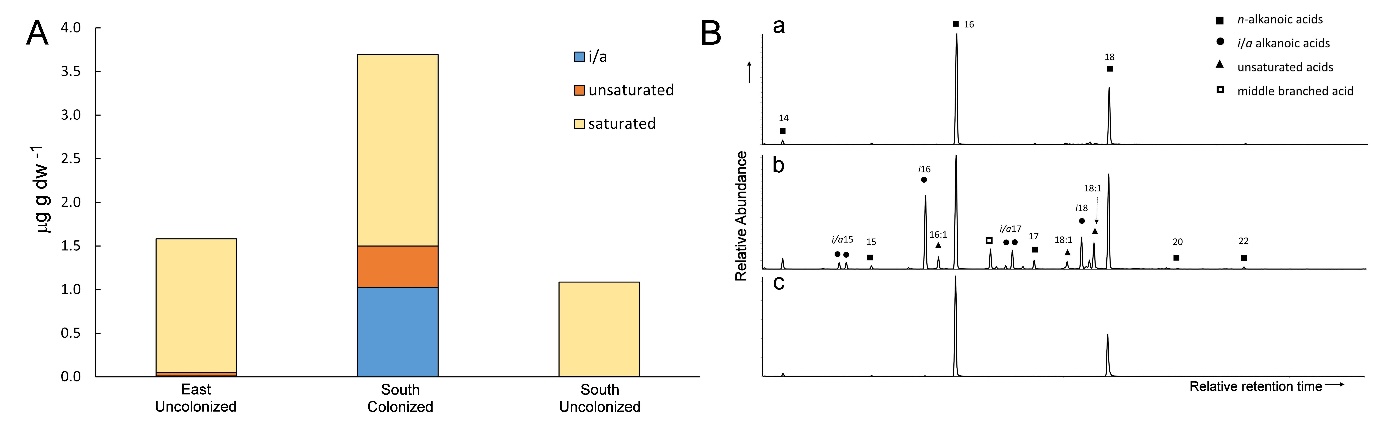


**Figure S10**. Relative abundance of lipid biosignatures in the acidic fraction of La Portada samples. A) Straight and saturated alkanoic acids, chains containing double bonds (unsaturated alkanoic acids), and terminally branched chains (i.e., containing a methyl group in position iso- or anteiso-, i/a). B) Mass chromatogram (ion m/z 74) of the acid fraction from La Portada samples (a, East Uncolonized; b, South Colonized; c, South Uncolonized). Black squares, dots, triangles, and white squares stand for linear and saturated (i.e., n-alkanoic acids), terminally branched (i.e., methyl group in penultimate or C-3 positions, that is iso or anteiso acids), unsaturated (i.e., containing double bonds), and mid-chain branched carboxylic acids (a.k.a. alkanoic acids). Numbers over the peaks indicate the number of carbons in the chain. Unsaturated acids are represented in the N:M format, where N indicates the number of carbons composing the chain and M the number of double bonds (e.g., 18:1 is a chain of 18 carbons with one double bond).

**Table S1**. XRD semiquantitative analysis of La Portada Cliff rock samples.


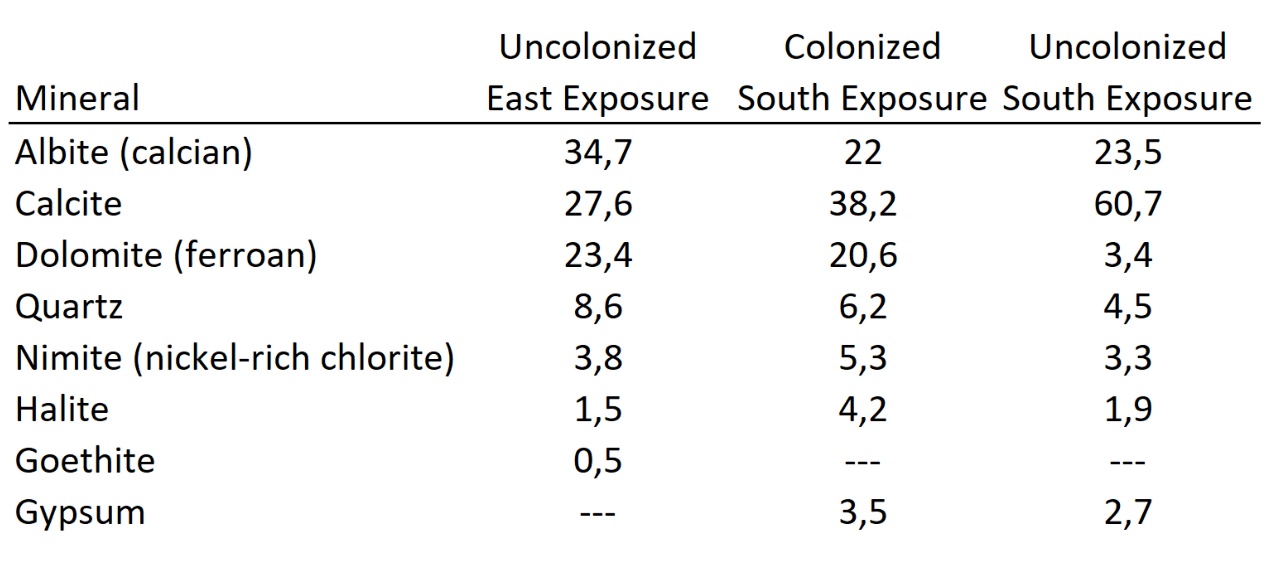


| Compounds^a^ | Uncolonized E | Colonized S | Uncolonized S |
| --- | --- | --- | --- |
| *i*C_14:0_ | n.d. | 0.029 | n.d. |
| C_14:0_ | 0.033 | 0.080 | 0.021 |
| *i*C_15:0_ | 0.001 | 0.045 | 0.001 |
| *a*C_15:0_ | 0.002 | 0.044 | 0.001 |
| C_15:0_ | 0.010 | 0.021 | 0.005 |
| *i*C_16:0_ | 0.001 | 0.523 | 0.003 |
| C_16:1ω7_ | n.d. | 0.088 | n.d. |
| C_16:1ω5_ | 0.002 | 0.006 | n.d. |
| C_16:0_ | 1.007 | 1.251 | 0.717 |
| 10-Mme16 | n.d. | 0.137 | n.d. |
| Br-Mme17 | n.d. | 0.020 | n.d. |
| *i*C_17:0_ | n.d. | 0.024 | n.d. |
| *a*C_17:0_ | n.d. | 0.134 | 0.001 |
| C_17:1ω7_ | n.d. | 0.023 | n.d. |
| C_17:0_ | 0.007 | 0.064 | 0.002 |
| 9-Mme17 | 0.012 | n.d. | n.d. |
| C_18:1ω5_ | n.d. | 0.070 | n.d. |
| 10-Mme17 | 0.004 | n.d. | n.d. |
| *i*C_18:0_ | 0.003 | n.d. | n.d. |
| 14-Mme17 | 0.003 | n.d. | n.d. |
| *a*C_18:0_ | 0.003 | 0.225 | n.d. |
| TetraMme16 | 0.010 | 0.021 | n.d. |
| C_18:1ω9_ | 0.022 | 0.066 | n.d. |
| C_18:1ω7_ | 0.013 | 0.213 | n.d. |
| C_18:0_ | 0.468 | 0.757 | 0.335 |
| C_19:1ω9_ | n.d. | 0.009 | n.d. |
| Cy_19:0_ | n.d. | 0.005 | n.d. |
| C_19:0_ | n.d. | 0.005 | n.d. |
| C_20:1ω9_ | n.d. | 0.001 | n.d. |
| C_20:0_ | 0.007 | 0.014 | n.d. |
| C_21:0_ | n.d. | n.d. | n.d. |
| C_22:0_ | 0.002 | 0.006 | n.d. |

**Table S2.** List of alkanoic acids detected in the samples from La Portada cliff microbialites, Chile (ug·g^-1^ dw).

n.d.: not detected.

^a^ The alkanoic acids are named as N:n, where N indicates the total number of carbon in the chain and n the number of double bonds. In polyunsaturated acids, the positions of the double bonds are indicated in the omega notation (i.e., respect to the end carbon). *Iso* (*i*N:0) and *anteiso* (*a*N:0) alkanoic acids are alkanoic acids with a methyl group in position N-1 or N-2, respectively. Mme: one methyl ramification and TetraMme four methyl ramifications in the acid chain, Cy: cyclopropyl acid.

**Table S3.** List of alcohols detected in the samples from La Portada cliff microbialites, Chile (ug·g^-1^ dw).

| Compounds^a^ | East, Uncolonized | South Colonized | South Uncolonized |
| --- | --- | --- | --- |
| 16:1 | n.d. | 0.014 | n.d. |
| 16 (I) | n.d. | 0.031 | n.d. |
| 16 (II) | 0.032 | 0.120 | 0.033 |
| 17 (I) | n.d. | 0.008 | n.d. |
| 17 (II) | n.d. | 0.006 | n.d. |
| 17 (II) | n.d. | 0.009 | n.d. |
| 18:1 (I) | n.d. | 0.009 | n.d. |
| 18:1(II) | n.d. | 0.287 | 0.041 |
| 18 (I) | n.d. | 0.043 | n.d. |
| 18 (II) | 0.015 | 0.555 | 0.042 |
| Dihydrophytol | 0.020 | 0.059 | n.d. |
| Phytol | n.d. | 0.026 | 0.005 |

n.d.: not detected.

^a^ Linear and saturated (*normal*) alkanols (*n*-alkanols) are named by simple numbers (N) that indicate the number of carbons in the chain; alkanols with a double bond are named as N:1. Notation : (I), and (II), indicates homologue compounds with the same chain length and double bond.
